# Single-cell profiling of T and B cell repertoires following SARS-CoV-2 mRNA vaccine

**DOI:** 10.1172/jci.insight.153201

**Published:** 2021-12-22

**Authors:** Suhas Sureshchandra, Sloan A. Lewis, Brianna M. Doratt, Allen Jankeel, Izabela Coimbra Ibraim, Ilhem Messaoudi

**Affiliations:** 1 Department of Molecular Biology and Biochemistry,; 2 Institute for Immunology, and; 3 Center for Virus Research, University of California, Irvine, Irvine, California, USA.; 4 Department fo Microbiology, Immunology and Molecular Genetics, University of Kentucky, Levington, Kentucky, USA.

**Keywords:** COVID-19, Vaccines, Adaptive immunity, T cell receptor, T cells

## Abstract

mRNA vaccines for SARS-CoV-2 have shown exceptional clinical efficacy, providing robust protection against severe disease. However, our understanding of transcriptional and repertoire changes following full vaccination remains incomplete. We used scRNA-Seq and functional assays to compare humoral and cellular responses to 2 doses of mRNA vaccine with responses observed in convalescent individuals with asymptomatic disease. Our analyses revealed enrichment of spike-specific B cells, activated CD4^+^ T cells, and robust antigen-specific polyfunctional CD4^+^ T cell responses following vaccination. On the other hand, although clonally expanded CD8^+^ T cells were observed following both vaccination and natural infection, CD8^+^ T cell responses were relatively weak and variable. In addition, TCR gene usage was variable, reflecting the diversity of repertoires and MHC polymorphism in the human population. Natural infection induced expansion of CD8^+^ T cell clones that occupy distinct clusters compared to those induced by vaccination and likely recognize a broader set of viral antigens of viral epitopes presented by the virus not seen in the mRNA vaccine. Our study highlights a coordinated adaptive immune response in which early CD4^+^ T cell responses facilitate the development of the B cell response and substantial expansion of effector CD8^+^ T cells, together capable of contributing to future recall responses.

## Introduction

The COVID-19 pandemic has spurred the rapid development of vaccines targeting SARS-CoV-2 that have garnered emergency-use authorizations from the FDA and are being widely distributed (1, 2). The Pfizer (BNT162b2) and Moderna (mRNA-1273) mRNA-based vaccines were the first to be approved and have proven to be safe and efficacious (94% effective) in adults and children over 12 years of age (3, 4). While the long-term (up to 6 months after dose 2) safety and efficacy of mRNA vaccines has been demonstrated, the mechanisms by which they elicit early cellular immune responses to SARS-CoV-2 remain poorly understood (5). In this study, we aimed to address two questions: What are the functional and transcriptomic responses of memory T and B cells to mRNA-based COVID-19 vaccination? And how does the vaccine response differ from that of an asymptomatic SARS-CoV-2 infection?

Recent studies have shown that development of robust neutralizing antibody and memory B cell responses requires both mRNA vaccine doses in SARS-CoV-2–naive individuals, while comparable humoral responses are generated with just 1 dose in convalescent individuals (6). However, the ratio of binding-to-neutralizing antibodies after vaccination was greater than that after infection (7). Additionally, most vaccinees had Th1-skewed T cell responses, where early T follicular helper (Tfh) cells and Th1 CD4^+^ responses correlate with effective neutralizing antibody responses after the first dose and CD8^+^ effector responses after the second dose (8). Furthermore, expanded T cell clones detected following vaccination were predominantly memory cells whereas those detected during infection were to effector cells with acute infection (9). Collectively, these observations suggest distinct T and B cell responses following vaccination in comparison to natural infection. Additional studies that integrate functional, transcriptional, and repertoire analysis of the memory immune cell response to COVID-19 mRNA vaccination are needed (8).

In this study, we assayed humoral and cellular responses to 2 doses of mRNA vaccine (14 days after dose 2) in 4 individuals and compared parallel changes in their immune repertoire with changes observed in 3 convalescent individuals who experienced asymptomatic/mild COVID-19 (~30 days after positive COVID test). A single dose of mRNA vaccines induced neutralizing titers at comparable levels as those seen following asymptomatic/mild SARS-CoV-2 infection. However, neutralizing titers in vaccinees increased several-fold following the second vaccine dose, exceeding those detected following asymptomatic/mild SARS-CoV-2 infection. Antigen-specific B cells were detected after the second dose. ScRNA-Seq analysis revealed an expansion of activated CD4^+^ T cells expressing transcription factors associated with of Th1 and Th17 subsets after vaccination. In line with these transcriptional findings, robust antigen-specific polyfunctional Th1 and Th17 responses were observed within CD4^+^ T cells in all vaccinated individuals. Although CD8^+^ T cell responses were weak and highly variable, effector memory (EM) CD8^+^ T cell clones were expanded in every individual following vaccination. TCR gene usage was variable reflecting the diversity of T cell repertoires and MHC polymorphism in the human population. Natural infection induced expansion of distinct CD8^+^ T cell clones, likely due to the recognition of a broader set of epitopes presented by the virus not seen in the mRNA vaccine.

## Results

### Humoral responses to SARS-CoV-2 mRNA vaccination.

**Figure 1:**
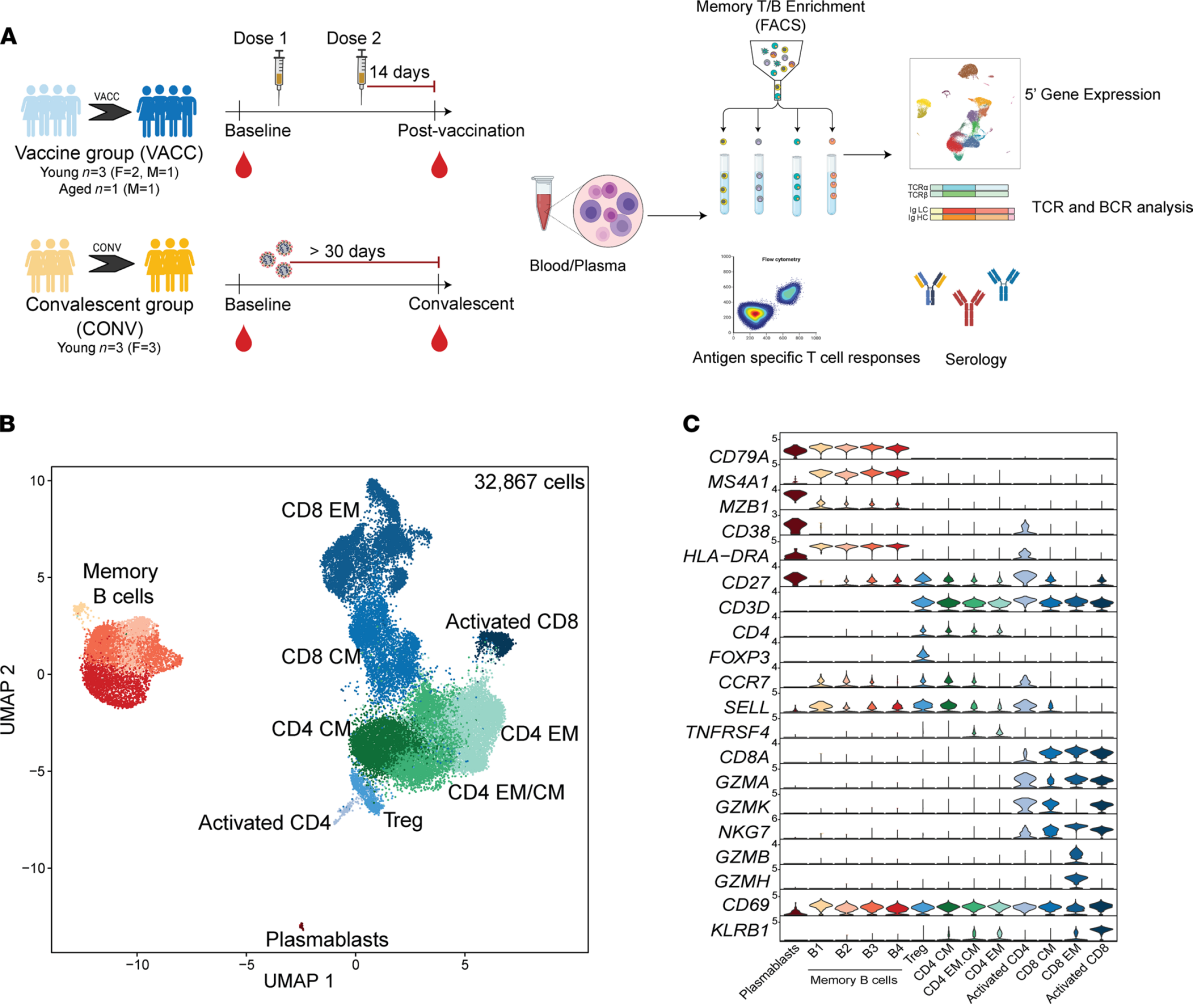
Immunological changes with SARS-CoV-2 mRNA vaccination. (**A**) Experimental design for the study. Blood was collected from SARS-CoV-2–naive individuals before vaccination, 2 weeks after dose 1, and 2 weeks after prime-boost vaccination (VACC group) or in SARS-CoV-2–exposed but asymptomatic individuals (CONV group) before and after convalescence. Immune phenotypes of PBMCs and antigen-specific T and B cell responses were measured using multicolor flow cytometry. Longitudinal serological responses to the vaccine were measured using ELISA and neutralization assays. Memory T and B cells from a subset of PBMC samples (*n* = 4/group for vaccine volunteers, *n* = 3/group for convalescent health care workers, matched) were profiled using scRNA-Seq at baseline (before vaccination) or after vaccination time points. (**B**) UMAP projection of 32,867 memory T and B cells with major subsets annotated. (**C**) Violin plots of key gene markers used for cluster annotations. Normalized transcript counts are shown on the *y* axis.

To comprehensively assess the cellular and humoral immune response to COVID-19 vaccination, we collected blood from SARS-CoV-2–naive volunteers prior to mRNA vaccination (baseline) and 2 weeks following prime-boost vaccination (postvaccination dose 2; *n* = 4) (Figure 1A). These responses were compared to those generated by individuals who experienced asymptomatic SARS-CoV-2 infection using longitudinal samples collected before (baseline) and approximately 30 days after exposure (convalescent, *n* = 3). Demographic and vaccine information are provided in Supplemental Table 1 (supplemental material available online with this article; https://doi.org/10.1172/jci.insight.153201DS1). Both infection and vaccination induced binding IgG and IgA (Supplemental Figure 1, A and B) and neutralizing antibodies (Supplemental Figure 1C), as early as 2 weeks following the first dose of vaccine; antibody levels increased several-fold following booster vaccination (Supplemental Figure 1C) and reached slightly higher levels than those achieved following asymptomatic/mild infection (*P* = 0.09). Given that full protection against the virus is achieved 2 weeks after the booster, we chose this time point for the additional analyses.

To specifically assess distinct memory responses, we sorted memory T and B cells and circulating plasmablasts from PBMCs before and 2 weeks after booster vaccination or approximately 30 days after SARS-CoV-2 infection (Supplemental Figure 1D) and performed 5′ single-cell RNA-Seq (scRNA-Seq) combined with parallel repertoire analysis (Figure 1A). Dimension reduction of 32,867 cells from 4 vaccinated and 3 convalescent individuals (Supplemental Figure 1E) by Uniform Manifold Approximation and Projection (UMAP) separated clusters of cells that were identified as regulatory T cells (*FOXP3*), EM, central memory (CM), and activated CD4^+^ and CD8^+^ T cells (Figure 1, B and C). CM and EM subsets were distinguished based on relative expression of *CCR7*, *TNFRSF4*, *GZMH/B*, *NKG7*, and *SELL* (encoding CD62L), whereas activated CD4^+^ T cells expressed high levels of *CD38* and *HLA-DR* and activated CD8^+^ T cells expressed high levels of *CD69* and *KLRB1* (Figure 1C). We also identified 4 subsets of memory B cells based on relative expression of *CD27*, *SELL*, and *CCR7*. A small cluster of plasmablasts was identified based on *MZB1* and *CD38* expression (Figure 1, B and C).

### B cell responses to vaccination and infection.

**Figure 2:**
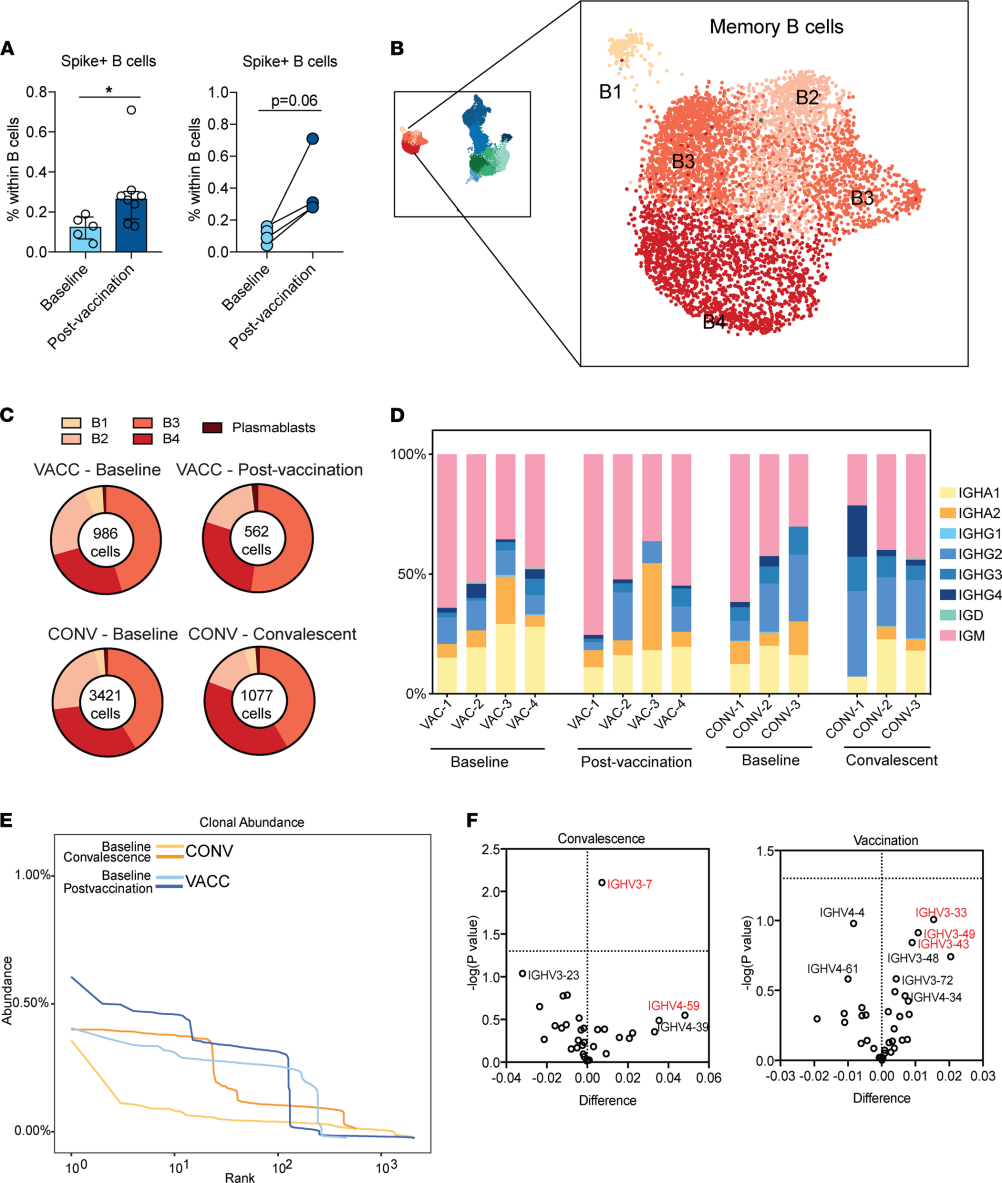
B cell adaptations following SARS-CoV-2 mRNA vaccination and infection. (**A**) Dot plots representing expansion of spike^+^ cells within total CD20^+^ B cells in PBMCs before and after vaccination (aggregate differences at baseline, *n* = 5, and following vaccination, *n* = 8, on the left; matched differences on the right, *n* = 4). PBMCs were incubated with biotinylated spike protein and fluorochrome-conjugated streptavidin, surface stained, washed, and analyzed using flow cytometry. Group differences were tested using unpaired *t* test with Welch’s correction (left) or paired *t* test (right). Error bars denote medians and interquartile ranges. (**B**) Magnified image of B cell subsets identified using scRNA-Seq. Data include samples from all 4 groups. (**C**) Pie chart quantifying B cell cluster frequencies after infection and vaccination. (**D**) Isotype distribution of productive B cell clones in vaccinated (*n* = 4) and convalescent (*n* = 3) individuals. Isotypes were determined based on the constant region of the clone. (**E**) Aggregate clonal abundance following vaccination and infection. (**F**) Volcano plots depicting heavy chain gene usage biases following convalescence (relative to preinfection baseline) or vaccination (relative to prevaccination baseline). The *x* axis represents the change in gene usage, and the *y* axis represents *P* value (–log_10_). *P < 0.05.

We next examined the B cell responses to vaccination. Both vaccination and asymptomatic infection resulted in the reduction of naive and expansion of memory B cells, with these changes being more prominent with natural infection (Supplemental Figure 2A). Importantly, antigen-specific (spike-specific) B cells were detected in circulation 2 weeks after prime-boost vaccination (Figure 2A and Supplemental Figure 2B). Examination of memory B cells using scRNA-Seq revealed 4 major clusters (Figure 2B) exhibiting distinct patterns of immunoglobulin genes (Supplemental Figure 2C): (a) a less mature cluster B1 expressing high levels of *IGHD* and *IGHM*; (b) cluster B2 expressing lower levels of *IGHM* but higher levels of *IGHA1*; (c) cluster B3 sharing features with B2 but also expressing *IGHG1* and *IGHG2*; and (d) cluster B4 the expressing the highest levels of *IGHG2* (Supplemental Figure 2C). Comparison of cluster proportions revealed a contraction of less mature clusters (B1 and B2) and expansion of mature clusters (B3 and B4) with both vaccination and infection (Figure 2C). Plasmablast proportions increased in 3 of 4 individuals following vaccination (*P* = 0.19) (Supplemental Figure 2D). We then compared gene expression profiles between memory B cells collected 2 weeks after vaccination and those in convalescent patients approximately 30 days after exposure. Genes upregulated (log_2_ [fold change] ≥ 0.4 and FDR ≤ 0.05) with vaccination relative to infection (*n* = 44) played a role in leukocyte activation, whereas those upregulated with infection (*n* = 96) enriched to additional GO terms, including cytokine production, apoptosis, cell adhesion, and type I IFN signaling (Supplemental Figure 2E).

B cell repertoire analysis resolved memory B cells into distinct isotypes (Figure 2D). Expansion of IgG^+^ cells was evident in a subset of individuals after vaccination and infection, whereas vaccination resulted in a significant reduction in IgA1^+^ memory B cells (*P* = 0.018) (Figure 2D). Despite this reduction, levels of spike-specific IgA increased with booster vaccination and infection (Supplemental Figure 1B). Clonal analysis of B cells revealed expansion of small-sized clones (10–100 cells) with both vaccination and infection, albeit to a lesser magnitude with vaccination (Figure 2E). Infection only was associated with expansion of larger B cell clones (>100 cells) (Figure 2E). Finally, gene usage analysis revealed preferential usage of heavy chain gene family IGHV3: IGHV3-7 and IGHV4-59 with convalescence and IGHV3-33, IGHV3-43, and IGHV3-49 with vaccination (Figure 2F).

### T cell adaptations with vaccination and convalescence.

**Figure 3:**
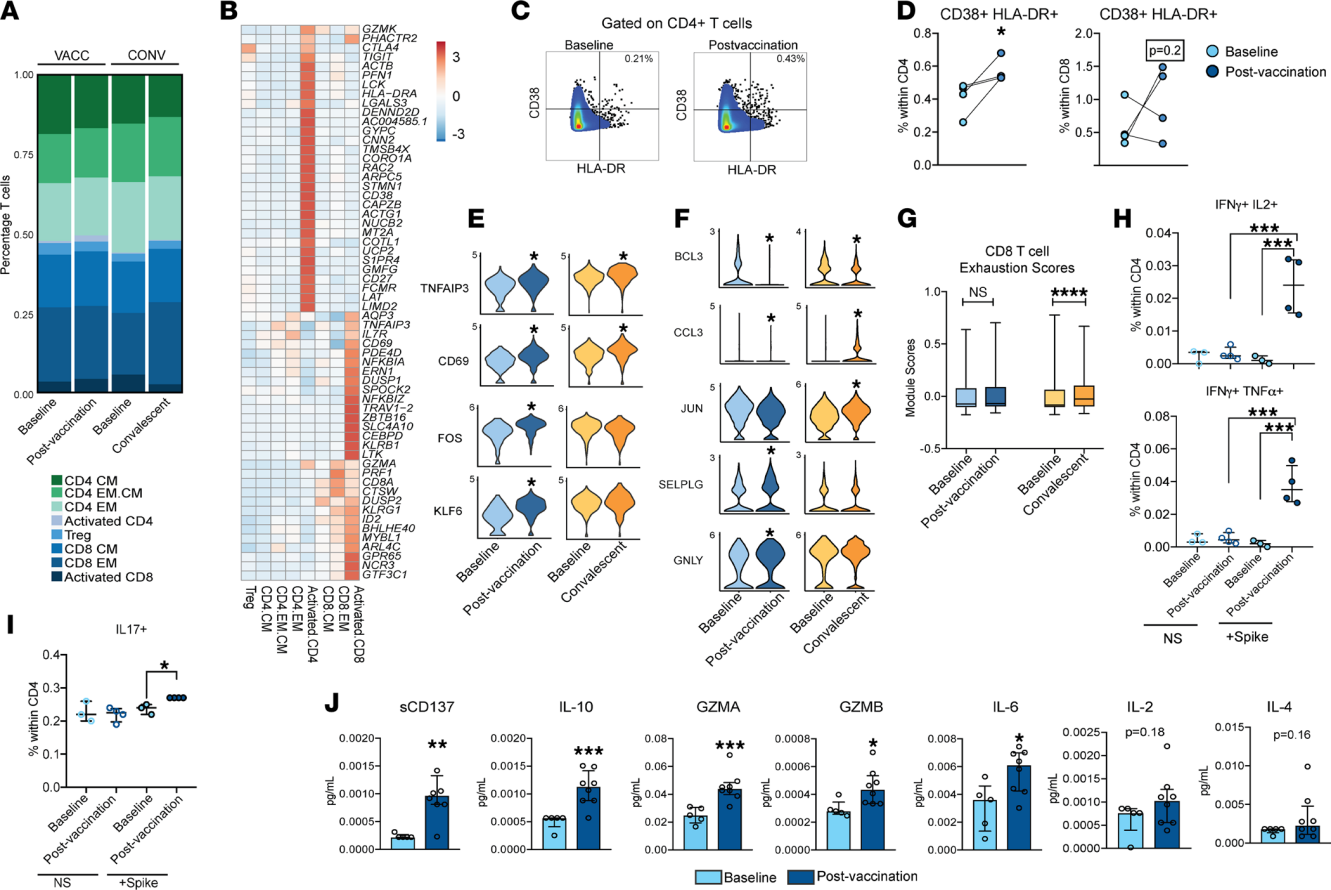
T cell adaptations with SARS-CoV-2 mRNA vaccination. (**A**) Stacked bar graph comparing the distribution of memory CD4^+^ and CD8^+^ T cells across each group, reported as percentage of total cells. (**B**) Clustered heatmap comparing aggregate top markers from each of the memory T cell clusters. Colors represent normalized transcript levels, ranging from low (in blue) to high (in red). (**C**) Gating strategy for identification of activated CD4^+^ and CD8^+^ T cells before and after vaccination. (**D**) Frequencies of CD38^+^HLA-DR^+^ CD4^+^ and CD8^+^ T cells following vaccination (*n* = 4/group). (**E** and **F**) Violin plots comparing key genes differentially expressed in (**E**) activated CD8^+^ and (**F**) CD8^+^ EM subsets either with convalescence and/or vaccination. (**G**) Box plot comparing exhaustion scores within CD8^+^ T cells with convalescence and/or vaccination. Lines indicate quartiles and median scores. (**H**) Polyfunctional CD4^+^ T cell (Th1) and (**I**) Th17 responses following overnight stimulation with SARS-CoV-2 spike–overlapping peptide pool, measured using intracellular cytokine staining at baseline (*n* = 3) and following vaccination (*n* = 4). (**J**) Secreted levels of soluble costimulatory molecule (sCD137), cytokines (IL-10, IL-6, IL-2, and IL-4), and effector molecules (granzyme A and granzyme B) in CD4^+^ T cells at baseline (*n* = 5) or following mRNA vaccination (*n* = 8). Two-way comparisons were tested using either paired test for matched comparisons or unpaired *t* test with Welch’s correction for group comparisons. Four-way comparisons were tested using 1-way ANOVA followed by Holm-Šidák multiple-hypothesis correction. Error bars denote medians and interquartile ranges. *P < 0.05; **P < 0.01; ***P < 0.001; ****P < 0.0001.

Next, we examined the effects of mRNA vaccination or natural infection on the distribution of memory T cell subsets. The CD4^+^ CM (Tcm) subset expanded with vaccination (*P* = 0.08) but not asymptomatic infection (Supplemental Figure 3A). No major changes in other CD4^+^ subsets (naive, Tem) or CD8^+^ subsets were detected with flow cytometry (Supplemental Figure 3, A and B). Single-cell analyses revealed an expansion of activated CD4^+^ T cells (Figure 3A) with vaccination that expressed relatively higher levels of *CD38* and *HLA-DRA* and cytotoxic molecules *GZMK* and *PRF1* (Figure 3B). On the other hand, frequencies of activated CD8^+^ T cells (expressing *CD69* and *KLRG1*) were comparable across groups (Figure 3, A and B). The expansion of activated HLA-DR^+^CD38^+^CD4^+^ T cells following vaccination was confirmed using flow cytometry (Figure 3, C and D). This expanded activated CD4^+^ T cell subset had elevated expression of Th1 transcription factor gene *TCF7* (encoding Tcf1) as well as *RORA* (encoding ROR-α) following vaccination (Supplemental Figure 3C). Interestingly, expression of *GATA3*, associated with Th2 cells, was reduced with both vaccination and infection (Supplemental Figure 3C). Activated CD8^+^ T cells exhibited increased expression of activation markers *CD69* and *TNFAIP3* with both infection and vaccination (Figure 3E). On the other hand, factors associated with memory development (*FOS*, *KLF6*) were upregulated with vaccination but not asymptomatic infection (Figure 3E). Within the CD8^+^ EM subset, both vaccination and infection resulted in the upregulation of *BCL3* (Figure 3F), which is essential for maximum IFN-γ secretion following secondary antigen stimulation. However, tissue-homing factor *SELPG* (encoding P selectin) and cytotoxic gene *GNLY* (encoding granulysin) were only induced with vaccination (Figure 3F). Finally, we examined CD8^+^ T cell exhaustion by scoring a core set of exhaustion-associated genes (*PDCD1* encoding PD-1, *HAVCR2* encoding Tim-3, *CD160*, *LAG3*, *CD244*, and *CTLA4*). This analysis revealed increased CD8^+^ T cell exhaustion with infection but not with vaccination (Figure 3G).

### Robust antigen-specific CD4^+^ T cell effector responses with vaccination.

We next interrogated antigen-specific CD4^+^ and CD8^+^ T cell responses following vaccination. PBMCs were stimulated with an overlapping peptides library covering the entire sequence of the spike protein for 24 hours, surface stained, fixed, and analyzed for cytokine production using flow cytometry (Supplemental Figure 3D). Spike-specific polyfunctional IFN-γ^+^IL-2^+^ and IFN-γ^+^TNF-α^+^ (Th1) CD4^+^, but not CD8^+^, T cells were evident 2 weeks after prime-boost vaccination (Figure 3H and Supplemental Figure 3E), in line with increased expression of *TCF7* (Supplemental Figure 3C). Additionally, vaccination induced Th17 responses in CD4^+^ T cells (Figure 3I), in agreement with increased expression of *RORA* (Supplemental Figure 3C). To gain a more comprehensive understanding of the effector T cell response induced by vaccination, we stimulated FACS-sorted CD4^+^ and CD8^+^ T cells with spike peptides for 24 hours and measured secreted factors using Luminex (Supplemental Figure 3F). CD4^+^ T cells secreted elevated levels of cytokines (IL-6, IL-10), cytotoxic molecules (granzyme A and granzyme B), and costimulatory factor (sCD137; soluble 4-1BB) (Figure 3J). However, IL-2 and IL-4 levels remained unchanged (Figure 3J), in line with a reduction of *GATA3*-expressing CD4^+^ T cells (Supplemental Figure 3C) and strongly suggesting that mRNA vaccines promote the development of a Th1/Th17 response. No significant production of immune mediators was noted by CD8^+^ T cells, except for perforin and modest levels of IFN-γ (Supplemental Figure 3G). Finally, enhanced secreted levels of apoptotic factor sFas was observed in both CD4^+^ and CD8^+^ T cells following vaccination (Supplemental Figure 3H).

### Vaccination and infection induce different T cell clonal expansions.

**Figure 4:**
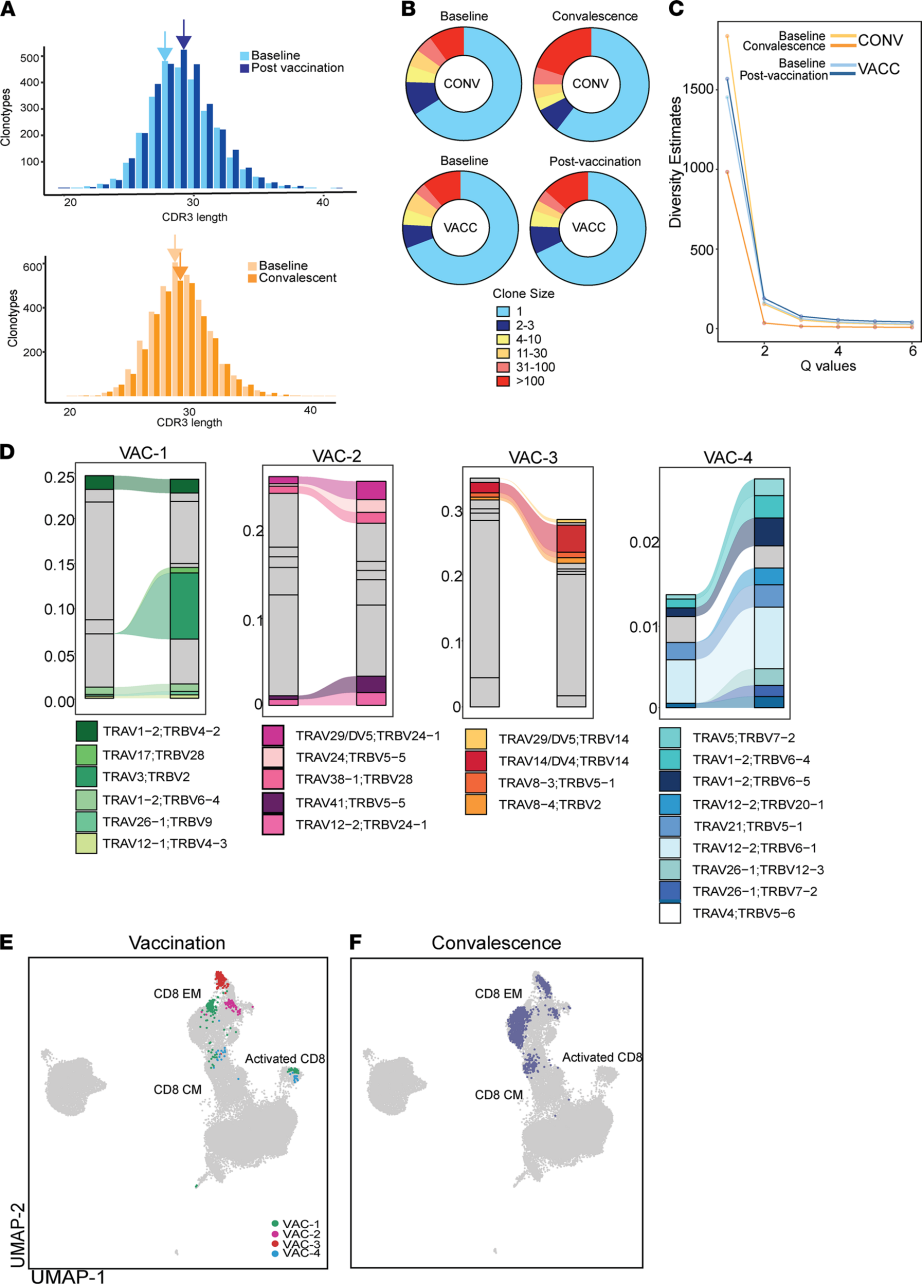
Clonal expansion of T cells following SARS-CoV-2 mRNA vaccination and infection. (**A**) CDR3 distribution of T cell clones following vaccination (top) and infection (bottom). Arrows highlight shifts in peak CDR3 lengths. (**B**) Pie chart representations of distribution of T cell clone sizes following vaccination and infection. (**C**) Diversity profiles of T cells following vaccination (*n* = 4, 2 time points) and infection (*n* = 3, 2 time points). The *y* axis represents Hill diversity, interpreted as the effective number of clonotypes within the data set. (**D**) Clonotype tracking in 4 volunteers for 2 weeks following the second dose of mRNA vaccine. Only the top 10 clones after vaccination with evidence of clonal expansion following vaccination are highlighted. (**E** and **F**) UMAP projection of the top 10 expanded clones following (**E**) vaccination (each participant is highlighted) and (**F**) convalescence.

Next, we compared the changes in T cell clonal dynamics with infection or vaccination. Changes in CDR3 length diversity and sequence diversity are a robust tool to monitor the T cell response to antigenic exposure. Differences in clonal dominance and size distribution could indicate preferential expansion of antigen-specific clones, and examination of these changes across multiple individuals provides insight into public and private TCR repertoires. Vaccination was associated with a shift toward increased CDR3 lengths (Figure 4A). Infection was associated with expansion of large clones (>100 cells), while vaccination induced expansion of primarily small-sized clones (2–3 cells) (Figure 4B and Supplemental Figure 4, A and B); however, these expansions were highly variable among the vaccinated individuals (Supplemental Figure 4B). Finally, both vaccination and infection were associated with a drop in clonal diversity; however, this drop was more dramatic following infection (Figure 4C).

We next examined biases in TCRα and TCRβ gene usage by comparing repertoire assignments after convalescence and vaccination with their respective baselines. Within TCRα, we observed limited overlap between vaccination and infection groups with a positive bias toward TRAV39 (*P* = 0.0003 with convalescence; *P* = 0.23 with vaccination), TRAV29/DV5 (*P* = 0.3 with convalescence; *P* = 0.09 with vaccination), TRAV21 (*P* = 0.02 with convalescence; *P* = 0.36 with vaccination) (Supplemental Figure 4C), TRBV10 (*P* = 0.1 with convalescence; *P* = 0.0.03 with vaccination), and TRBV12 (*P* = 0.09 with convalescence; *P* = 0.2 with vaccination) (Supplemental Figure 4D). However, convalescence and vaccination preferentially enriched distinct TCRs: TRAV29/DV5; TRBV5-1 and TRAV29/DV5; and TRBV6-5 with convalescence (Supplemental Figure 4, E and F) and TRAV29/DV5; TRBV11-2 and TRAV29/DV5; TRBV7-9 and TRAV12-2; and TRBV6-2 with vaccination (Supplemental Figure 4G). We observed diverse patterns of clonal expansion with few clones that expanded dramatically in Vac-1, Vac-2, and Vac-3, while several smaller clones with limited expansion were detected in Vac-4 (Figure 4D). Finally, the T cell clones that expanded with vaccination or convalescence occupied distinct space within the UMAP (Figure 4, E and F). Interestingly, the top expanded clones following vaccination were mostly CD8^+^ EM with smaller involvement of CD8^+^ CM and activated CD8^+^ T cells (Figure 1B and Figure 4E). However, expanded clones within convalescent individuals included both CD8^+^ EM and CD8^+^ CM subsets (Figure 1B and Figure 4F).

## Discussion

The establishment of immunity against SARS-CoV-2 has become a central focus of current research efforts. Natural immunity following infection and vaccine-generated immunity provide two different pathways to immunity against the disease. mRNA vaccines have demonstrated significant protection against severe COVID-19 disease. Findings from human trials of Pfizer/BioNTech and Moderna vaccines suggest 95% maximal protection within 1 to 2 months after the second vaccine dose, including against several circulating variants of concern (10, 11). Recommendations from the CDC indicate that individuals are not fully protected until 2 weeks after the second dose of vaccine (12). In this study, we investigated the cellular changes in circulating T and B cells induced by SARS-CoV-2 mRNA vaccination in 4 individuals before vaccination and 2 weeks after the second dose and compared clonal adaptations in individuals who developed natural immunity against the virus.

The presence of neutralizing antibodies is currently used as a surrogate indicator of immunity. Both mRNA vaccines induce potent and durable neutralizing antibodies as early as 10 days (6) and last up to 8 months after the first dose of vaccination (13). Our data suggest that while neutralizing titers after the first dose are comparable to those observed in recovered individuals, levels of neutralizing titers become significantly higher in the vaccinated group following the booster. However, both vaccinees and convalescent individuals shared several key B cell adaptations. For example, flow analysis revealed a reduction in naive but expansion of memory B cells in both groups. Additionally, scRNA-seq analysis revealed a reduction in IgA^+^ (*IGHA1*) memory B cells following vaccination, as recently described in individuals who have recovered from COVID-19 (14). These cells are less likely to be receptor-binding domain (RBD) specific, given that the expanded antigen-specific B cells following vaccination are IgG^+^ (6). However, elevation in RBD-specific IgA in plasma was observed with both vaccination and infection. A more recent study has demonstrated the presence of RBD-specific soluble IgA in the saliva from patients with COVID-19 and vaccinated individuals, with moderate neutralizing capacity, but persisting up to 6 months after the second dose (15). Collectively, these findings suggest that an intramuscular mRNA vaccine induces mucosal antibody response in both saliva and blood. Within PBMCs from SARS-CoV-2–naive individuals, we observed a modest expansion of plasmablasts (in 3 of 4 subjects), and a significantly elevated presence of spike-reactive B cells (in all 4 individuals) following vaccination, suggesting establishment of durable memory and potential recall responses to infection. Finally, while signatures of immune activation were upregulated in memory B cells following vaccination and infection, gene expression markers associated with regulation of type I interferon signaling, cytokine production, and apoptotic signaling were more pronounced in convalescent patients. These observations support the fact that while vaccination results in B cell evolution that lasts for a few weeks, memory B cells following natural infection continue to evolve over several months (16).

Clonal analysis of memory B cells revealed expansion of small-sized clones in recovered individuals and, to a lesser extent, in vaccinated individuals. This is in line with recent reports of limited evidence of somatic hypermutation in spike-binding memory B cell clones in vaccinated individuals 1 week after second dose (6). We argue that this is likely because our sampling captured very early events in B cell responses and assessment at later time points would reveal more clones that emerge from prolonged B cell evolution within the germinal center (GC), as observed in recovered individuals (17, 18). Supporting this hypothesis, a recent study analyzing lymph node aspirates of vaccinated individuals observed S-binding GC B cells and plasmablasts for at least 15 weeks after first dose, further expanding after second dose (19). Interestingly, circulating IgG- and IgA-secreting spike-specific plasmablasts peaked 1 week after the second dose and then declined, becoming undetectable 3 weeks later (19). This is in line with our findings, where we observed increased, but weak, enrichment of CD27^+^CD38^++^ plasmablasts in blood 2 weeks following the second dose. Finally, mRNA vaccine–induced GC B cells are at near peak frequencies for at least 12 weeks after the second dose (19), suggesting that changes in B cell repertoire at least 2–3 months following vaccination should be assessed in the future.

The characteristics of cellular immunity induced by mRNA vaccines remain unclear. Early studies testing mRNA vaccine efficacy have demonstrated robust antigen-specific Th1 responses following 2 doses of vaccines (8). Our single-cell analyses suggest an expansion of activated CD4^+^ T cells (CD38^+^HLA-DR^+^) that are skewed toward a Th1 and Th17 phenotype, as recently demonstrated in the nasal mucosa of mRNA-vaccinated individuals (20). On the other hand, frequencies of activated CD8^+^ T cells remained unchanged after vaccination. Moreover, CD8^+^ T cells in convalescent, but not vaccinated, individuals exhibit signs of exhaustion (characterized by increased expression of *PD-1*, *TIM-3*, *LAG-3*) despite the fact that these individuals experienced asymptomatic/mild disease.

In the presence of lower neutralizing antibodies following the first vaccine dose, rapid induction of SARS-CoV-2–specific CD4^+^ T cells in SARS-CoV-2–naive individuals has been argued to play a role in protection after the first dose of vaccine (9). In contrast, antigen-specific CD8^+^ T cells have been reported to develop gradually and reach maximal levels only after the second dose (9). Indeed, overnight stimulation of PBMCs with overlapping 15-mers covering full-length spike protein revealed enrichment of polyfunctional CD4^+^ T cells in all vaccinated individuals following the booster dose. Cytokine analysis of stimulated CD4^+^ T cells also suggested a robust Th1/Th17 response and a lack of Th2 response to spike peptides. In contrast, cytokine-producing CD8^+^ T cells were observed in only 2 of the 4 individuals, and effector responses to spike peptides were weak, suggesting a delay in development of effector CD8^+^ T cell response. Our findings are in line with those of early efficacy studies and recent follow-up studies, both 1 week after booster and up to 7 months after dose 1, where the magnitude of CD8^+^ T cell responses (measured using both AIM assay and intracellular cytokine responses) was both variable and several-fold lower in comparison to CD4^+^ T cell responses (8, 9). To some extent this is not surprising, given that the most immunodominant epitopes recognized by CD8^+^ T cells in patients with COVID-19 are contained in ORF1 and not spike protein (21). Nevertheless, whether the strength of early CD4^+^ T cell response and/or variability in CD8^+^ T cell responses in vaccinees is predictive of durable neutralizing titers and/or long-term memory B cell responses is yet to be evaluated. Interestingly, persistence of antigen-specific CD4^+^ and CD8^+^ T cells 7 months after the first dose (13) supports the hypothesis that mRNA vaccine induces durable CD4^+^ and CD8^+^ T cell responses capable of contributing to future recall responses.

Both vaccination and convalescence enriched T cell clones with longer CDR3s, though this shift was more prominent with vaccination. Moreover, infection induced a sharper drop in diversity of T cell repertoire compared with vaccination. Surprisingly, clonal tracking analysis revealed expansion of EM CD8^+^ T cells in all individuals, albeit the magnitude of expansion was very weak in 1 aged individual included in this study. This is in contrast to very limited expansion within CD4^+^ T cells following the second dose of vaccination. We posit that analysis of T cell clones as early as 2 weeks after dose 1 would allow for better assessment of clonal expansion within CD4^+^ T cells, which we might have missed given our sampling window. Alternatively, future studies will have to evaluate CD4^+^ T cell clones at various time points following in vitro enrichment of antigen-specific clones. Expansion of EM CD8^+^ T cell clones following vaccination, however, is in line with what has been reported with natural infection (14, 22, 23). Despite the substantial expansion of CD8^+^ T cell clones, frequency of S-specific CD8^+^ T cells was small and variable. This discrepancy could be due to bystander activation. Expanded T cell clones with vaccination and infection occupied distinct space on single-cell maps, highlighting differences in the breadth of the epitopes recognized in vaccinated compared with infected individuals (21).

Interestingly, early postboost induction of spike-specific memory B cells has been shown to correlate negatively with age following mRNA vaccination (6). Incidentally, the individual with the lowest CD8^+^ T cell expansion and the lowest levels of RBD-binding antibodies in this study is also the oldest individual in our cohort (Vac-4). This is in line with data from clinical trials showing lower neutralizing responses after a 100 μg dose and faster waning of the response following a low dose (25 μg) of mRNA-1273 (13, 24) in the elderly. Furthermore, natural infection has been shown to impair SARS-CoV-2–specific priming of CD8^+^ T cells in the elderly (25, 26). Whether that defect extends to vaccine induced early CD4^+^ T cell responses or subsequent CD8^+^ T cell expansion remains to be seen. Our study, however, was limited by a small sample size to draw definitive conclusions on weakening of vaccine responses in the aged. Moreover, it is still unclear what magnitude of neutralizing response confers protective immunity to the virus.

The timing and role of Tfh CD4^+^ responses following mRNA vaccination is less understood. The presence of S-specific circulating Tfh cells in patients with COVID-19 has been shown to positively correlate with plasma neutralizing activity (27–29). Interestingly, in vaccinated individuals, circulating Tfh responses 1 week after the first dose have been shown to correlate with postboost neutralizing antibodies (9). Due to limited number of PBMCs available to us, we were unable to perform profiling of circulating Tfh cells. However, a more recent study has demonstrated that frequency of S-reactive Tfh cells peaks 1 week after prime boost in the blood but diminishes thereafter (30). Tfh responses in the lymph node, however, persist at higher levels in the lymph node even 6 months after immunization (30). Taken together, these observations highlight the role of Tfh cell responses in both blood and GCs following vaccination.

Limitations of our study include small sample size and restriction to participants receiving mRNA vaccine. Given that we obtained few memory B cells from each individual, we were unable to perform rigorous somatic hypermutation analysis at the single-cell level. Finally, future studies will have to focus on long-term protection (both cellular and humoral) of 2 doses of mRNA vaccine against the numerous variants of SARS-CoV-2 and mechanisms of decline in quality of protection (if any) in the elderly.

## Methods

### Experimental design.

All participants in this study were healthy, and none reported any comorbidities. All vaccines (VACC group) received either the Pfizer (BNT162b2) or the Moderna (mRNA-1273) mRNA-based vaccines. Blood was collected at 3 time points: before vaccine baseline, 2 weeks after primary vaccine (dose 1), and 2 weeks after prime-boost vaccination (dose 2). Blood collected at 2 weeks after primary vaccine (dose 1) was used only for serological experiments. Baseline and postvaccination samples were analyzed for cellular and humoral response to the vaccine. For convalescent individuals (CONV group), blood samples collected before exposure to SARS-CoV-2 (baseline) and approximately 30 days after convalescence were included in the analysis. These individuals experienced asymptomatic/mild COVID-19. Detailed characteristics of participants and experimental breakdown by sample are provided in Supplemental Table 1.

### Plasma and PBMCs isolation.

Whole blood samples were collected in EDTA vacutainer tubes. PBMCs and plasma samples were isolated after whole-blood centrifugation at 1200*g* for 10 minutes at room temperature in SepMate tubes (STEMCELL Technologies). Plasma was stored at –80°C until analysis. PBMCs were cryopreserved using 10% DMSO/FBS and Mr. Frosty Freezing containers (Thermo Fisher Scientific) at –80°C and then transferred to a cryogenic unit 24 hours later until analysis.

### Measuring antibody responses.

RBD end-point titers were determined using standard ELISA, and plates were coated with 500 ng/mL SARS-CoV-2 Spike-protein RBD (GenScript). Heat-inactivated plasma (1:50 in blocking buffer) was added in 3-fold dilutions. Responses were visualized by adding HRP anti-human IgG or IgA (BD Pharmingen) followed by the additional of Phenylenediamine dihydrochloride (Thermo Fisher Scientific). ODs were read at 490 nm on a Victor3 Multilabel plate reader (Perkin Elmer). Batch differences were minimized by normalizing to positive control samples run on each plate.

Focus reduction neutralization titer was measured using heat-inactivated plasma serially diluted (1:3) in HyClone DMEM supplemented with 10 mM of HEPES buffer. The diluted plasma was preincubated with SARS-CoV-2 (100 PFU) for 1 hour before being transferred onto Vero E6 cells (ATCC, C1008) seeded in a 96-well plate, followed by overlay using 1% methylcellulose (MilliporeSigma). After 24 hours, the medium was carefully removed, and the plates were fixed. The number of infected foci was determined using anti–SARS-CoV-2 Nucleocapsid antibody (Novus Biologicals, NB100-56576) and HRP anti-rabbit IgG antibody (BioLegend). Plates were developed using True Blue HRP substrate (Sigma-Aldrich) and imaged on an ELISPOT reader (Autoimmun Diagnostika Gmbh). Each plate included a positive and a negative control. The half-maximum inhibitory concentration was calculated by nonlinear regression analysis using normalized counted foci on Prism 7 (Graphpad Software). 100% of infectivity was obtained by normalizing the number of foci counted in the wells derived from the cells infected with SARS-CoV-2 virus in the absence of plasma.

### Adaptive immune phenotyping.

Frozen PBMCs were thawed, washed in FACS buffer (2% FBS, 1 mM EDTA in PBS), and counted on TC20 (Bio-Rad) before surface staining using the following panel: CD4 (BioLegend, OKT4), CD8b (Beckman Coulter, 2ST8.5H7), CD28 (eBioscience, CD28.2), CD95 (eBioscience, DX2), CD20 (eBioscience, 2H7), IgD (BioLegend, IA6-2), CD27 (BioLegend, M-T271), and CD38 (Stemcell Technologies, AT1). Dead cells were excluded using the Ghost Dye Red 710 (Tonbo). T cell phenotyping was conducted using an additional panel of antibodies — CD4 (Biolegend, OKT4), CD8b (Beckman Coulter, 2ST8.5H7), CCR7 (Biolegend, G043H7), CD45RA (Miltenyi Biotec, T6D11), CD38 (Tonbo Biosciences, HIT2), CD27 (Biolegend, O323), HLA-DR (Biolegend, L243), CD69 (Biolegend, FN50), and PD-1 (Biolegend, EH12.2H7). All samples were acquired on the Attune NxT acoustic focusing cytometer (Life Technologies). Data were analyzed using FlowJo v10 (TreeStar).

### Antigen-specific T cell responses.

Approximately 1 × 10^6^ PBMCs were stimulated with 1 μg of the SARS-CoV-2 peptide pool 5 (S protein) or anti-CD3 (positive control) in 96-well plates for 24 hours at 37°C and 5% CO_2_. Plates were spun and surface stained using an antibody cocktail containing CD4 (OKT4, BioLegend), CD8b (Beckman Coulter, 2ST8.5H7), CD28 (eBioscience, CD28.2), and CD95 (eBioscience, DX2). Cells were washed, fixed and stained intracellularly using TNF-γ (eBioscience, MAB11), IL-2 (Biolegend, MQ1-17H12), IL-17A (eBioscience, 64DEC17) and IFN-γ (eBioscience, 4S.B3). Samples were analyzed on Attune NxT Flow cytometer (Thermo Fisher Scientific). Data were analyzed on FlowJo (BD Biosciences).

Approximately 5 × 10^4^ CD4^+^ and CD8^+^ T cells were sorted and stimulated with 1 μg of the SARS-CoV-2 peptide pool 5 (S protein) or anti-CD3 (positive control) for 16 hours at 37°C and 5% CO_2_. Plates were spun, and supernatants collected and stored in –80°C. Immune mediators in supernatants were measured using a Milliplex MAP Human CD8^+^ T cell 17-plex magnetic bead panel measuring GM-CSF, sCD137, IFN-γ, IL-10, granzyme A, granzyme B, IL-13, sFas, IL-2, IL-4, IL-5, sFasL, MIP-1α, MIP-1β, TNF-γ, and perforin per the manufacturer’s instructions and run on Magpix (Luminex Corp.). Standard curves were fit using 5P-logistic regression on XPonent software (Luminex Corp.).

### Antigen-specific B cells.

To detect antigen-specific B cells, approximately 5 × 10^5^ PBMCs were stained with 100 ng full-length biotinylated spike protein (Sino Biological) preincubated with Streptavidin-BV510 (Biolegend) at a 2:1 ratio for 1 hour at 4°C to ensure maximum staining quality before surface staining with CD20-FITC (Biolegend, 2H7) for an additional 30 minutes. Streptavidin PE (Biolegend) was used as a decoy probe to gate out SARS-CoV-2 nonspecific streptavidin binding. Samples were washed twice and resuspended in 200 μL FACS buffer before being analyzed on Attune NxT (Life Technologies).

### FACS for repertoire analysis.

Cryopreserved PBMCs from each person (*n* = 4 for pre- and postvaccine samples; *n* = 3 for baseline and convalescent samples) were thawed, washed, and stained with 1 μg/test cell-hashing antibody (BioLegend, TotalSeq C0251, C0254, C0256, C0260; clones LNH-95, 2M2) for 30 minutes at 4°C. Samples were washed 3 times in ice-cold PBS supplemented with 2% FBS and sorted on the FACSAria Fusion (BD Biosciences) with Ghost Dye Red 710 (Tonbo Biosciences) for dead cell exclusion and then CD4, CD8, CD28, CD95, CD38, CD27, and IgD to sort memory CD4^+^ T cells, CD8^+^ T cells, memory B cells, and plasmablasts. Live, sorted cell populations were counted in triplicates on a TC20 Automated Cell Counter (Bio-Rad) and pooled into 4 samples (before vaccine, after vaccine, baseline, and convalescent).

### 5′ scRNA-Seq.

Pooled cells were resuspended in ice-cold PBS with 0.04% BSA in a final concentration of 1800 cells/μL. Single-cell suspensions were then immediately loaded on the 10X Genomics Chromium Controller with a loading target of 26,000 cells. Libraries were generated using the Chromium Next Gem Single Cell 5′ Reagent Kit v2 (Dual Index) per the manufacturer’s instructions, with additional steps for the amplification of HTO barcodes and V(D)J libraries (10X Genomics). Libraries were sequenced on Illumina NovaSeq with a sequencing target of 30,000 reads per cell RNA library, 5000 reads per cell HTO barcode library, and 5000 reads per cell for V(D)J libraries.

### Single-cell RNA-Seq data analysis.

Raw reads were aligned and quantified using the Cell Ranger Single-Cell Software Suite with Feature Barcode addition (version 4.0, 10X Genomics) against the GRCh38 human reference genome using the STAR aligner. Downstream processing of aligned reads was performed using Seurat (version 4.0). Droplets with ambient RNA (cells fewer than 200 detected genes), dying cells (cells with more than 20% total mitochondrial gene expression), and cells expressing both a TCR and BCR clonotype were excluded during initial QC. Data normalization and variance stabilization were performed on the integrated object using the *NormalizeData* and *ScaleData* functions, where a regularized negative binomial regression corrected for differential effects of mitochondrial gene expression levels. The *HTODemux* function was then used to demultiplex donors and further to identify doublets, which were then removed from the analysis. Dimension reduction was performed using the *RunPCA* function to obtain the first 30 principal components, followed by integration using Harmony. Clusters were visualized using the UMAP algorithm, as implemented by Seurat’s *RunUMAP* function. Cell types were assigned to individual clusters using the *FindMarkers* function, with a fold-change cutoff of at least 0.4. A list of cluster-specific markers identified in this study is provided in Supplemental Table 2. Module scores for CD8^+^ T cell exhaustion were calculated using the *AddModuleScor*e function, aggregating expression for the following genes: *PDCD1*, *LAG3*, *HAVCR2*, *CD244*, *LAYN*, *CD160*, and *CTLA4*. Differential expression analysis was performed with MAST using default settings in Seurat. All disease comparisons were performed relative to healthy donors of corresponding age groups. Only statistically significant genes (log_10_[fold change] cutoff ≥ 0.25; adjusted *P* ≤ 0.05) were included in downstream analysis.

### TCR and BCR analysis.

TCR and BCR reads were aligned to VDJ-GRCh38 ensembl reference using Cell Ranger 4.0 (10X Genomics) generating sequences and annotations, such as gene usage, clonotype frequency, and cell-specific barcode information.

As an additional QC, only cells with one productive α and one productive β chain were retained for downstream analyses. CDR3 sequences were required to have length between 5 and 27 amino acids, start with a C, and not contain a stop codon. Cells with both TCR and BCR (<0.1%) assignments were excluded from the analysis and all downstream analysis was performed using the R package immunarch. Data were first parsed through *repLoad* function in immunarch and clonality examined using the *repExplore* function. Family and allele level distributions of TRA and TRB genes were computed using the *geneUsage* function. Diversity estimates (Hill numbers) were calculated using the *repDiversity* function, and tracking of abundant clonotypes was performed using *trackClonotype* function.

Clonal assignments based on heavy and light chains were determined using the change-o package in the Immcantation portal. Briefly, the heavy chain data were clonally clustered separately into their correct clonal groups assigned based on light chain data, removing cells associated with more than one heavy chain. Germline sequences were reconstructed using IgBlast. Gene usage, isotype abundance, and clonotype abundance were calculated using the Alakazam package in the Immcantation portal.

### Data availability.

The data sets supporting the conclusions of this article are available on NCBI’s Sequence Read Archive under BioProject PRJNA767017.

### Statistics.

Data sets were first tested for normality. All pairwise comparisons for readouts before/after vaccine and infection were tested using parametric paired 2-tailed *t* test. For comparisons involving multiple groups, differences were tested using 1-way ANOVA, followed by Holm-Šidák multiple-comparisons tests. *P* values of less than or equal to 0.05 were considered statistically significant. Values between 0.05 and 0.1 are reported as trending patterns.

### Study approval.

This study was approved by the University of California, Irvine, Institutional Review Boards. Informed consent was obtained from all enrolled individuals.

## Author contributions

SS, SAL, and IM conceived and designed the experiments. SS, SAL, BD, ICI, and AJ conducted experiments and interpreted data and SS, SAL, and BD analyzed the data. SS, SAL, and IM wrote the paper The order of co–first authors was determined based on contributions to revision. All authors have read and approved the final draft of the manuscript.

## Supplementary Material

Supplemental data

Supplemental table 1

Supplemental table 2

## Version 1 12/22/2021

Electronic publication
