# Supplementary material for: Single-cell profiling of T and B cell repertoires following SARS-CoV-2 mRNA vaccine
Source: JCI Insight. 2021 Dec 22;6(24):e153201. doi: 10.1172/jci.insight.153201 (PMC8783687; doi:10.1172/jci.insight.153201)
Supplement: Supplemental data [file jciinsight-6-153201-s114.pdf]

Supplemental Figure 1

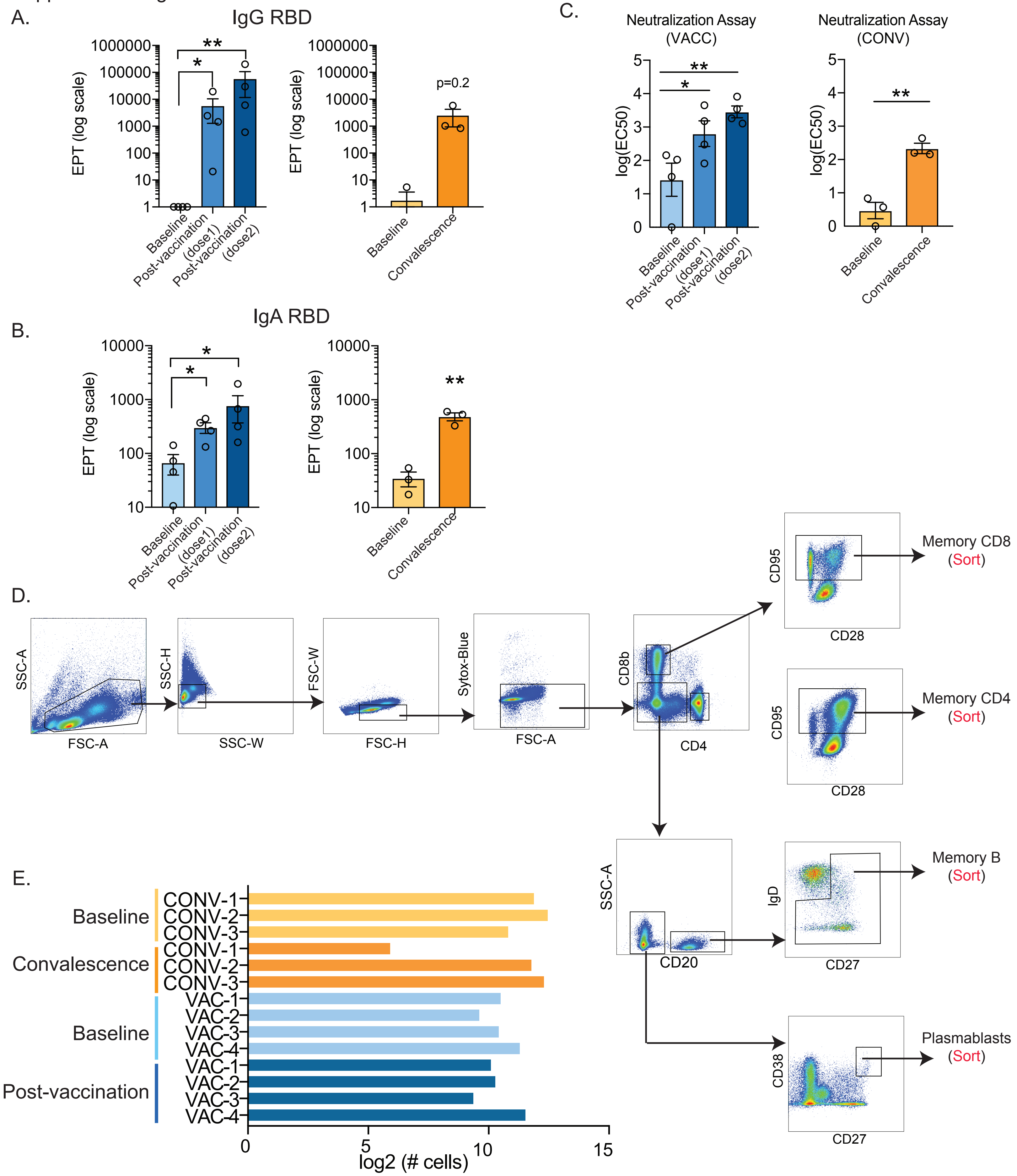

Supplemental Figure 1: Experimental design and humoral response to vaccination and infection

(A-B) Bar plots comparing (A) IgG and (B) IgA Endpoint titers before and after vaccination (two weeks post dose 1 and dose 2, n=4/group) or asymptomatic infection (n=3/group). (C) Bar plots comparing antibody neutralization of SARS-CoV-2 virus (IC50) before and after vaccination (two weeks post dose 1 and dose 2) and before and after asymptomatic infection. (D) Gating strategy for enrichment of memory T and B cells for single cell RNA and repertoire analysis. Cells were surface hashed using Total-Seq A antibodies and sorted based on surface CCR7 and CD45RA (for memory T cells), CD27 and IgD (for memory B cells), and CD27 and CD38 (for plasmablasts). (E) Sample wise breakdown of numbers of single cells recovered from each subject and used for downstream dimension reduction and clustering.Two-way comparisons were tested using parametric paired t-test [p-values: *- p<0.05; **-p<0.01]

Supplemental Figure 2

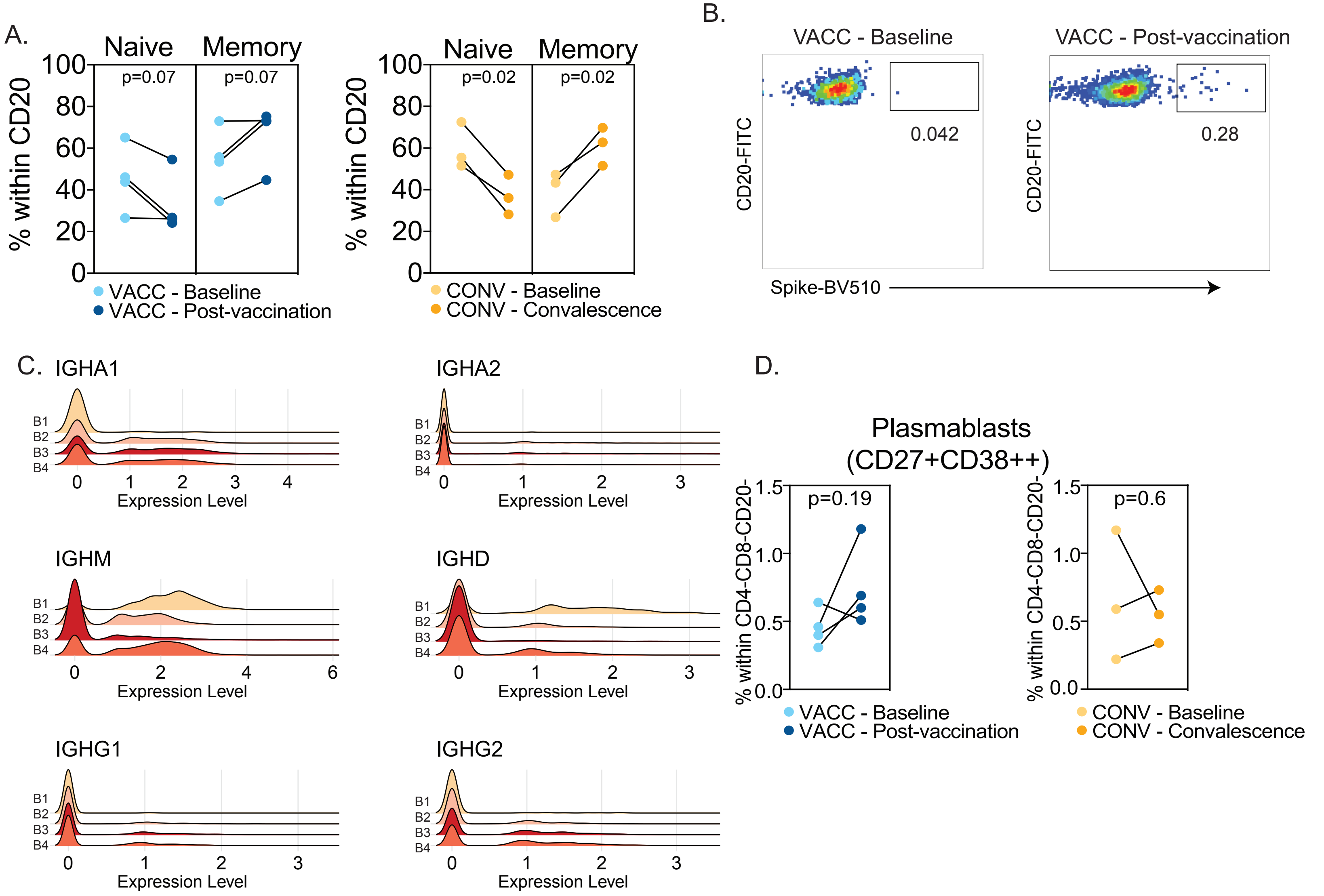

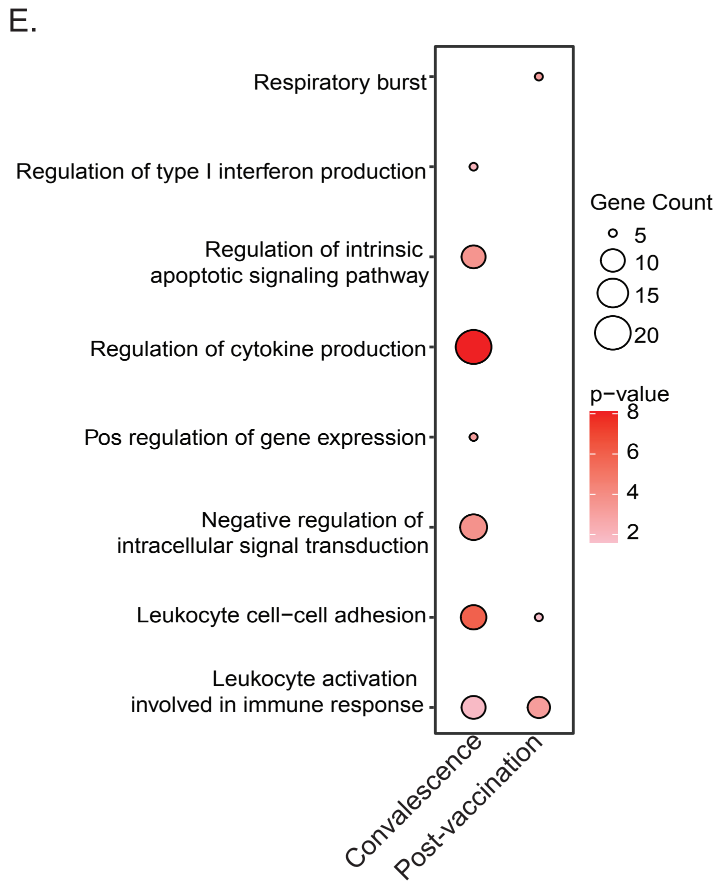

Supplemental Figure 2: B cell adaptations with mRNA vaccination and infection.
(A) Frequencies of naive and memory B cells before and after vaccination (left) vs. before and after asymptomatic infection (right).(B) Representative plots for identification of Spike+ events within gated B cells. (C) Ridge plots of immu - noglobulin genes across four memory B cell clusters identi - fied using single cell RNA sequencing. (D) Frequencies of plasmablasts before and after vaccination (left) vs. before and after asymptomatic infection (right). Differences were measured assessed using paired T-test. (E) Bubble plots representing two-way functional enrichment of genes up-regulated with infection or vaccination in a direct compar - ison of memory B cells post-vaccination vs. convalescence. Size of the bubble represents the number of genes mapping to the term whereas color represents level of statistical significance.

Supplemental Figure 3

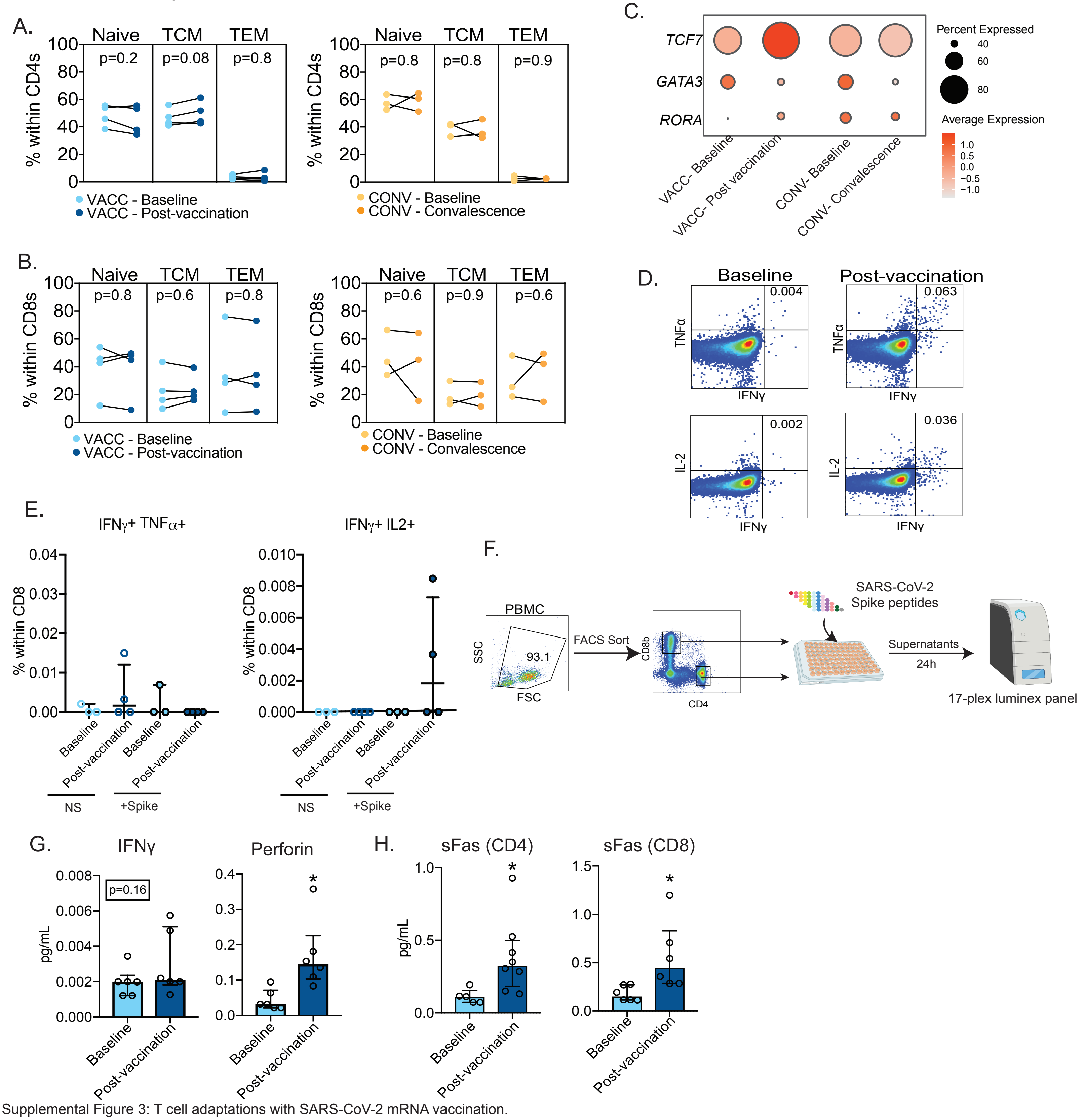

Supplemental Figure 3: T cell adaptations with SARS-CoV-2 mRNA vaccination.

(A) Frequencies of (A) CD4 and (B) CD8 T cells and their subsets before (n=4) and after vaccination (n=4). (C) Bubble plot representing expression of Th1, Th2, and Th17 associated transcription factors in the activated CD4 T cell cluster. Size of the bubble represents percentage of cells expressing the transcript, whereas color is indicative of relative expression. (D) Gating strategy for identification of cytokine producing cells following in vitro stimulation of PBMC with SARS-CoV-2 overlapping peptides. (E) Polyfunctional CD8 T cell responses following overnight SARS-CoV-2 spike peptide stimulation measured using intracellular cytokine staining and flow cytometry at baseline (n=3) and following mRNA vaccination (n=4). (F) Experimental design for measuring effector T cell responses following vaccination. Sorted CD4 and CD8 T ells from baseline (n=5) and post vaccination (n=8) samples were stimulated with SARS-CoV-2 overlapping spike peptides for 24 hours. Supernatants were collected and analyzed using Luminex. (G) Secreted levels of IFNγ and Perforin by CD8 T cells and (H) sFAS molecule by CD4 and CD8 T cells following peptide stimulation before and after mRNA vaccination. Two-way comparisons were tested using either parametric paired t-test for matched comparisons or unpaired t-test with Welch's correction for group comparisons. Four way comparisons were tested using one-way ANOVA followed by Holm SIdak's multiple hypothesis correction [p-values: *- $p<0.05$]

Supplemental Figure 4

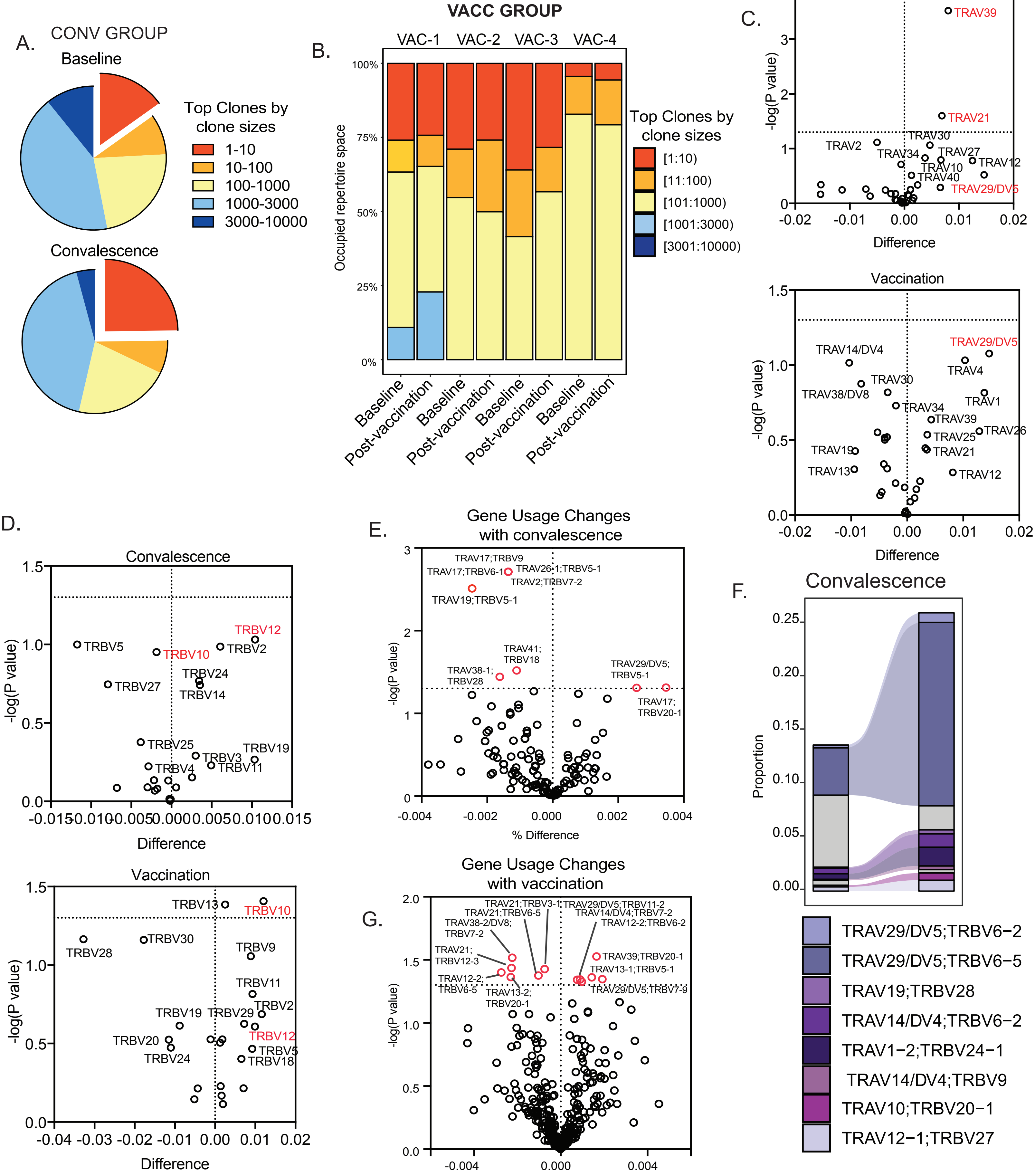

Supplemental Figure 4: T cell clonal expansion with SARS-CoV-2 mRNA vaccine. (A) Pie charts demonstrating aggregate clonal space occupied by top clones (by size) before and after convalescence. (B) Stacked bars showing distribution of top clones before and after vaccination in four volunteers. (C-D) Volcano plots depicting (C) TCRα and (D) TCRβ gene usage following convalescence (top panel, relative to pre-infection baseline) or vaccination (bottom panel, relative to pre vaccination baseline) at the gene family level. (E-F) Volcano plots depicting gene usage changes (TCRαβ combinations) following (E) convalescence (top panel, relative to pre-infection baseline) or (F) vaccination (bottom panel, relative to pre vaccination baseline). X axis represents the change in gene usage and Y axis represents p-value (-log10). Statistical differences were tested using multiple t-tests. (G) Expanded T cell clones following convalescence. Only the top 10 clones with evidence of clonal expansion following SARS-CoV-2 infection are highlighted.
